# Multicenter cross-calibration of I-123 metaiodobenzylguanidine heart-to-mediastinum ratios to overcome camera-collimator variations

**DOI:** 10.1007/s12350-014-9916-2

**Published:** 2014-06-19

**Authors:** Kenichi Nakajima, Koichi Okuda, Mana Yoshimura, Shinro Matsuo, Hiroshi Wakabayashi, Yasuhiro Imanishi, Seigo Kinuya

**Affiliations:** 1 Department of Nuclear Medicine, Kanazawa University Hospital, 13-1 Takara-machi, Kanazawa, 920-8641 Japan; 2 Department of Physics, Kanazawa Medical University, Uchinada, Japan; 3 Department of Radiology, Tokyo Medical University, Tokyo, Japan; 4 Department of Radiology, Suzuka Central General Hospital, Suzuka, Japan

**Keywords:** Metaiodobenzylguanidine (MIBG) imaging, standardization, heart-to-mediastinum ratio, calibration phantom, collimator

## Abstract

**Background:**

The heart-to-mediastinum ratio (HMR) of ^123^I-metaiodobenzylguanidine (MIBG) showed variations among institutions and needs to be standardized among various scinticamera-collimator combinations.

**Methods:**

A total of 225 phantom experiments were performed in 84 institutions to calculate cross-calibration coefficients of HMR. Based on phantom studies, a conversion coefficient for each camera-collimator system was created, including low-energy (LE, n = 125) and a medium-energy (ME, n = 100) collimators. An average conversion coefficient from the most common ME group was used to calculate the standard HMR. In clinical MIBG studies (n = 52) from three institutions, HMRs were standardized from both LE- and ME-type collimators and classified into risk groups of <1.60, 1.60-2.19, and ≥2.20.

**Results:**

The average conversion coefficients from the individual camera-collimator condition to the mathematically calculated reference HMR ranged from 0.55 to 0.75 for LE groups and from 0.83 to 0.95 for ME groups. The conversion coefficient of 0.88 was used to unify HMRs from all acquisition conditions. Using the standardized HMR, clinical studies (n = 52) showed good agreement between LE and ME types regarding three risk groups (κ = 0.83, *P* < .0001, complete agreement in 90%, 42% of the patients reclassified into the same risk group).

**Conclusion:**

By using the reference HMR and conversion coefficients for the system, HMRs with various conditions can be converted to the standard HMRs in a range of normal to low HMRs.

## Introduction

I-123 metaiodobenzylguanidine (MIBG) has been used in patients with chronic heart failure, ischemic heart disease, and cardiomyopathy. The most widely accepted application, however, is in patients with heart failure.^1^,^2^ More than 20-year experiences in this field has been accumulated in Japan, and use of MIBG in prognostic evaluation is described in Japanese Circulation Society’s Nuclear Cardiology Guidelines.^3^ The neurological application of MIBG has also become common, in particular in patients with Lewy-body diseases.^4^,^5^

In most of the MIBG studies, the quantification method was essential in differentiating normal and abnormal sympathetic activity, and also high-risk and low-risk groups. The heart-to-mediastinum ratio (HMR) was a simple ratio of the heart and background, and generally good reproducibility has been reported in a single center analysis.^6^,^7^ When multiple centers are involved in a study, however, there are some preferences for the location of regions of interest (ROIs), which potentially cause variations among institutions and published studies. More importantly, HMR based on the medium-energy (ME) collimator showed higher values than that based on low-energy (LE) collimators.^8^ The nomenclature of collimators is classified into two major groups of LE and ME, but the camera vendors have created various types of collimators depending on the purpose in order to achieve good balance among resolution, sensitivity, and applicable energy range. The low-medium energy (LME) collimator is one of the examples created to cover the higher energy scatter portion of the ^123^I energy spectrum, in accordance with the widely used ^123^I-labeled radiopharmaceutical in Japan.

We have already made a phantom for MIBG planar imaging to cross-calibrate two acquisition conditions.^9^ As an extension of this idea, the purposes of this study were to accumulate MIBG data for the HMR from common vendors, and to establish the cross-calibration method among various camera and collimator combinations. Our hypothesis in this study is that all camera-collimator combinations can be unified to a standard HMR, so that comparison among multiple centers and the previous studies can be practically performed. The validity was also tested in clinical studies.

## Methods

### Phantom Design

Details of the phantom were written elsewhere.^9^ In brief terms, since the purpose of this phantom was to standardize the HMR among different collimator types by minimizing effects of septal penetration and Compton scatter, we tried to simplify the structure as much as possible, in order to calculate the same HMR using planar images (Taisei Medical, Co. Ltd, Osaka, Japan; Hokuriku Yuuki, Co. Ltd, Kanazawa, Japan). Each organ part, namely, heart, mediastinum, lung, and liver, was designed so that the radioactivity was distributed uniformly in the organ regions. The size of the phantom was 380 mm in width and length, and each organ was flat with a constant concentration. The thickness of each organ was adjusted by changing the number of acrylic slices. The acrylic slices, 5 mm in thickness, were pasted with various numbers and orders. The upper and lower slices were 10 mm in thickness. Four HMRs from anterior and posterior views were obtained from two types of the phantom.

### A Phantom Experiment

^123^I-MIBG of 111 MBq in 4,450 mL was prepared and filled into the two phantoms. Since all organ parts were connected as one compartment, no adjustment of radionuclide concentration for each organ part was required. A 3-cm acrylic plate was placed over the phantom when imaging was performed. The 256 matrix images were acquired from the anterior and posterior views for 3-10 minutes, which was a situation comparable to clinical MIBG imaging. The energy was centered at 159 keV with a 20% window. Hospitals using a 15% window also measured HMRs with this condition. The experiments were performed using 225 conditions in 84 institutions (see “Appendix”).

### A Mathematical Reference Value of HMRs

HMRs were mathematically calculated in these models, assuming the linear attenuation coefficient (µ) of ^123^I for water as 0.147 cm^−1^. The standard equation for attenuation, that is exponential of (−µ*x*), where *x* was thickness of attenuation, was used. For calculation purpose, slices were divided into 0.05 mm slices, and the summation of the count was calculated using Mathematica software (version 9, Wolfram Research, Inc., Champaign, IL). The mathematical reference HMR was the attenuation corrected HMR, while Compton scatter and septal penetration of gamma rays were not included. The reference HMR was 3.50 and 2.60 for the type 1 phantom, and 1.80 and 1.55 for the type 2 phantoms.^9^

### Cross Calibrations

**Figure 1:**
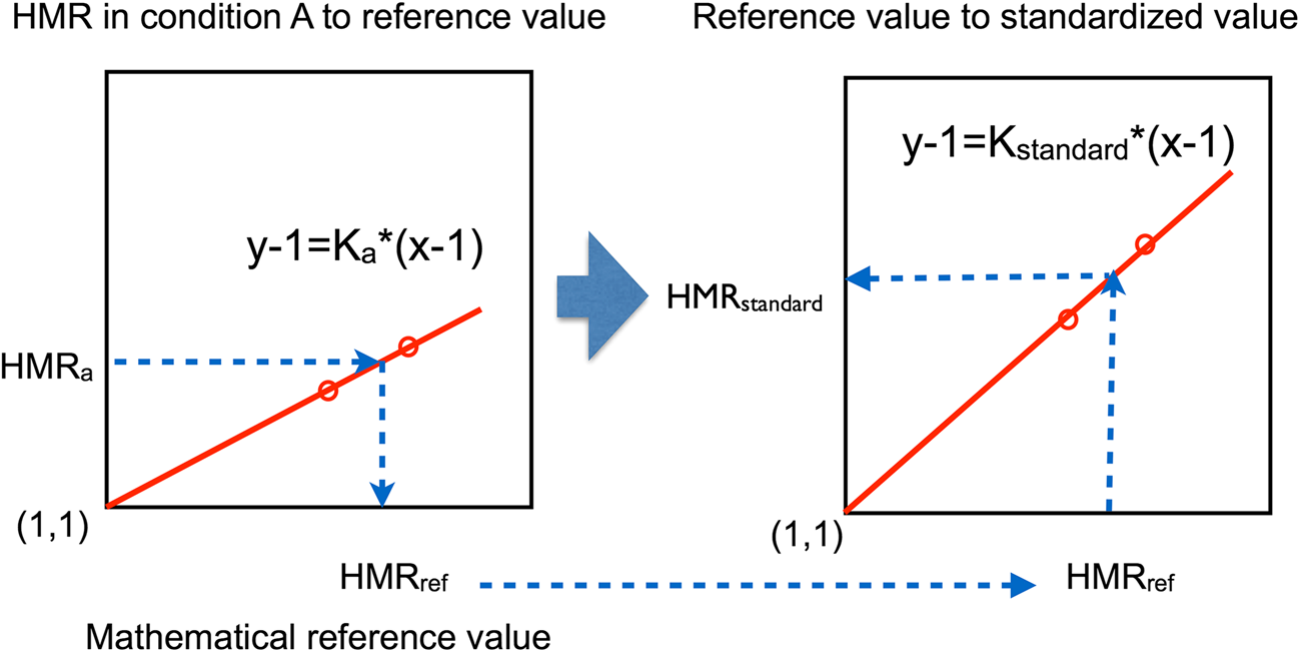
Conversion of HMR from the condition A (HMRa) to the reference value (HMRref), and to the standard value (HMRstandard). In this study, *K*_standard_ of 0.88 is used as the conversion coefficient, which is an average coefficient of common ME collimators. *Asterisk* denotes multiplication

A calibration method from LE-type collimator to ME-type collimator comparable values was already described.^10^ In this study, 4 or 2 HMRs from 2 phantoms types (anterior and posterior views for each) were plotted to the reference values (Figure 1). A linear regression equation was calculated using the formula of *y* − 1 = *K* * (*x* − 1) (* denotes multiplication), in which the line always passes on the coordinate (1,1). The first step was to convert the HMR with LE-collimator to the reference value (HMR_ref_) using coefficient *K*_a_, which is the slope of the regression line in condition A. The second step was to convert from the HMR_ref_ to a standardized HMR (HMR_standard_) using the *K*_standard_. The *K*_standard_ was defined as average *K* values for typical ME collimators. The conversion coefficient of HMR from LE to the standardized condition was identical to *K*_standard_/*K*_a_. The rationale for this conversion to the common ME-type is based on practical consideration, so that most of the users of the ME-collimator can use their routine HMRs.

### Clinical MIBG Imaging

Anterior MIBG images were obtained with a 256 × 256 matrix format for 3-5 minutes. In Hospital A, Prism 2000 with a LEHR collimator (Picker, Co. Ltd., Tokyo, Japan) and E.CAM Signature with LMEGP collimators (Siemens Japan Co. Ltd., Tokyo) were used (n = 12). In Hospitals B, GCA-9300A three-detector gamma cameras with LEHR (Toshiba, Tochigi, Japan) and LMEGP collimators (Siemens Japan Co. Ltd., Tokyo, Japan) were used (n = 10). In Hospital C, E.CAM systems with LEHR and LMEGP (Toshiba, Tochigi, Japan) were used (n = 15). In the Hospitals A and B, acquisition energy was set 159 keV with a 20% window, and with a 15% window in Hospital C according to institutional routine conditions. We did not change their preference for routine acquisition conditions. In Hospitals A and B, only a delayed image at 3 hours was obtained with two LE and ME conditions, and in Hospital C both 20-minute (early) and 3-hour (delayed) images were obtained. Thus, a total of 52 studies were obtained by both LE- and ME-type conditions in 37 patients (16 males and 21 females, aged 71 ± 10 years). Indication of the MIBG study was not specified in this technological validation study. However, the indications included ischemic and non-ischemic cardiac diseases suspicious of having heart failure and neurological diseases as Parkinson disease or syndrome, Alzheimer disease, and dementia with Lewy bodies.

HMRs were calculated using a semiautomatic ROI setting software.^11^ In the software algorithm, the heart region was set as a circle after manually pointing to the center of the heart, and a rectangular region was determined in the upper mediastinum, in which the width was 10% of the body, and the height was the upper 30% of the mediastinum. All the data were anonymized and processed in each hospital, and the calculated data were sent to Kanazawa University. Informed consent was obtained from all patients in each hospital. Ethical committees or comparable institutional regulation approved the study.

### Statistics

The data are shown as mean ± standard deviation. A difference among groups was examined by one-way analysis of variance and Student’s *t* test. The linear regression equation of the HMRs between two conditions was calculated by the least square method. When the HMRs were classified into three risk groups, considering that the average HMR in the normal databases was 2.8 and the lower limit was 2.2, thresholds of ≥2.20 (normal range or higher) and 1.60-2.19 (low risk) and <1.60 (high risk) were used. These thresholds were generally in agreement with the risk classifications used in heart failure patients.^1^-^3^,^12^,^13^ Contingency table analysis was performed and degree of agreement was tested. *P* values <0.05 were considered significant. The statistics software JMP version 10 (SAS Institute Inc., Cary, NC, USA) was used, and mathematical calculation was based on Mathematica 9 (Wolfram Research Inc., Champaign, IL, USA).

## Results

### Number of Experiments and Participating Institutions

A total of 225 experiments were performed in 84 institutions, including 7 camera vendors of Siemens (n = 71), GE (n = 56), Toshiba (n = 50), Shimadzu (n = 23), Philips (n = 19), Hitachi/Philips (n = 5), and ADAC (n = 1). Collimator types were divided into two major groups of LE (n = 125) and ME (n = 100). The LE groups included high-resolution (LEHR), general-purpose (LEGP), all-purpose (LEAP), general-all-purpose (LEGAP), extended LE general-purpose (ELEGP), and cardiac high-resolution (CHR). The ME group included low-medium-energy general-purpose (LMEGP), general-purpose (MEGP), general-all-purpose (MEGAP), low penetration (MELP), and high-energy general-purpose (HEGP). The nomenclature of collimators depended on manufacturers’ specifications.

### HMRs Measured in Four Phantom Conditions

**Figure 2:**
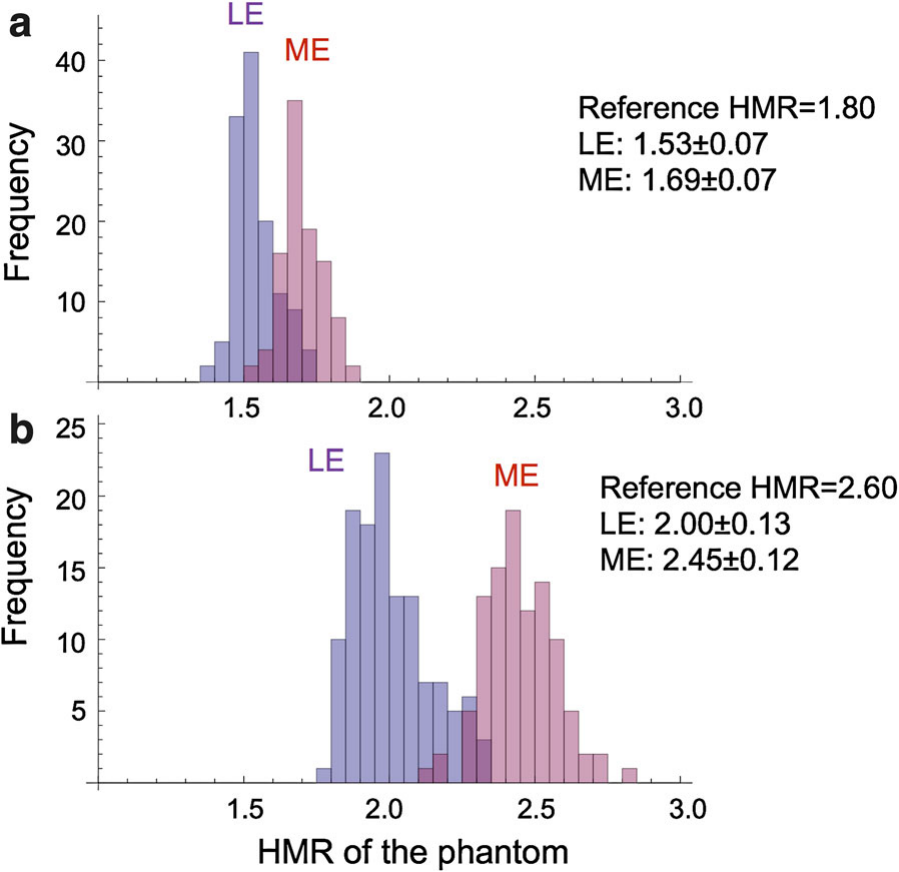
Distribution histograms of HMRs using phantoms with the reference HMR of 1.80 (**a**) and 2.60 (**b**). While the ME group shows higher values than the LE group, overlaps are observed

When HMRs were obtained using four phantom conditions, the LE-collimator group showed lower values compared with the ME-collimator group. For the phantom of HMR 1.55, LE-collimator (n = 125) and ME-collimator (n = 100) groups showed 1.40 ± 0.06 and 1.51 ± 0.06 (*P* < .0001). Similarly, the phantoms of HMR 1.80, 2.60, and 3.50 showed 1.53 ± 0.07 and 1.69 ± 0.07 (*P* < .0001), 2.00 ± 0.13 and 2.45 ± 0.12 (*P* < .0001), and 2.43 ± 0.19 and 3.10 ± 0.17 (*P* < .0001), for LE and ME groups, respectively. Distribution of the mathematical HMR of 1.8, 2.6 is shown as examples (Figure 2). While both LE and ME groups differed significantly, overlap of histogram distributions was also observed.

### Conversion Coefficients Determined by 4 and 2 Data Points

When cross-calibration equations passing on the (*x*, *y*) coordinate of (1,1) were made based on all 4 points and higher 2 points, the distribution of coefficients showed high correlation between two methods: (slope from the 2 points) = −0.0197 + 1.027 × (slope from the 4 points) (*R* ^2^ = 0.997, *P* < .0001). We therefore used coefficients from the upper two points in the following analyses.

**Figure 3:**
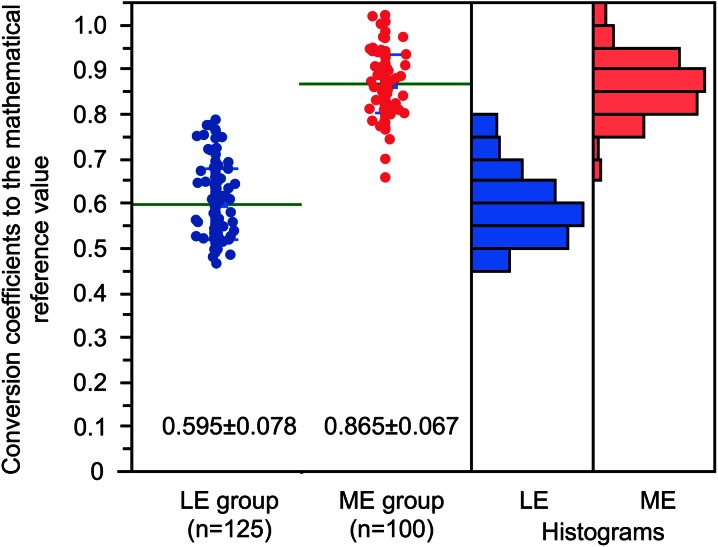
Conversion coefficients to the reference values in LE- and ME-collimator groups. *Bars* denote average values, and histogram distributions are also shown. Although a significant difference was observed between the mean values of LE and ME groups, an overlap was also noted

The conversion coefficients to the reference value are summarized according to the main collimator names, namely 5 LE subgroups and 3 ME subgroups (Table 1). Since the ELEGP collimator showed two separate distributions between old (2002-2004) and new (2008-2013) types due to modification of specification, it was divided into two subgroups. The average conversion coefficients were 0.55 for LEHR, 0.65 for LEGP/AP, 0.83 for LMEGP, and 0.88 for MEGP, and the highest was 0.95 for MELP/HEGP types. The difference among subgroups was highly significant (*F* ratio 214, *P* < .0001). When the conversion coefficient was divided into two groups, the average values were 0.595 ± 0.078 for the LE group and 0.865 ± 0.067 for the ME groups (*F* ratio 750, *P* < .0001) (Figure 3).

**Table 1:** Conversion coefficients to mathematical reference values

Collimator	LE or ME	N	Mean	SD	Lower 95%	Upper 95%	Vendor and number
LEHR	LE	73	0.55	0.05	0.54	0.56	GE (15), Siemens (25), Toshiba (22), Shimadzu (8), Philips/Hitachi (3)
CHR	LE	9	0.55	0.02	0.53	0.57	Philips/Hitachi (9)
LEGP/AP	LE	25	0.65	0.04	0.63	0.66	GE (10), Siemens (3), Shimadzu (7), Philips/Hitachi (4), ADAC (1)
ELEGP (old)	LE	4	0.62	0.03	0.57	0.67	GE (4)
ELEGP (new)	LE	14	0.75	0.03	0.73	0.76	GE (14)
LMEGP	ME	46	0.83	0.05	0.81	0.85	Siemens (25), Toshiba (21)
MEGP/GAP	ME	40	0.88	0.05	0.86	0.89	GE (13), Siemens (9), Toshiba (3), Shimadzu (8), Philips/Hitachi (7)
MELP/HEGP	ME	14	0.95	0.04	0.92	0.98	Siemens (9), Toshiba (4), Hitachi (1)

*LE*, Low-energy; *ME*, medium-energy; *CHR*, cardiac HR; *GP*, general-purpose; *AP*, all-purpose; *GAP*, general-all-purpose; *ELE*, extended LE; *LME*, low-medium energy; *LP*, low penetration; *HEGP*, high-energy general-purpose.

### HMRs from LE and ME Collimators and Effect of Standardization

**Figure 4:**
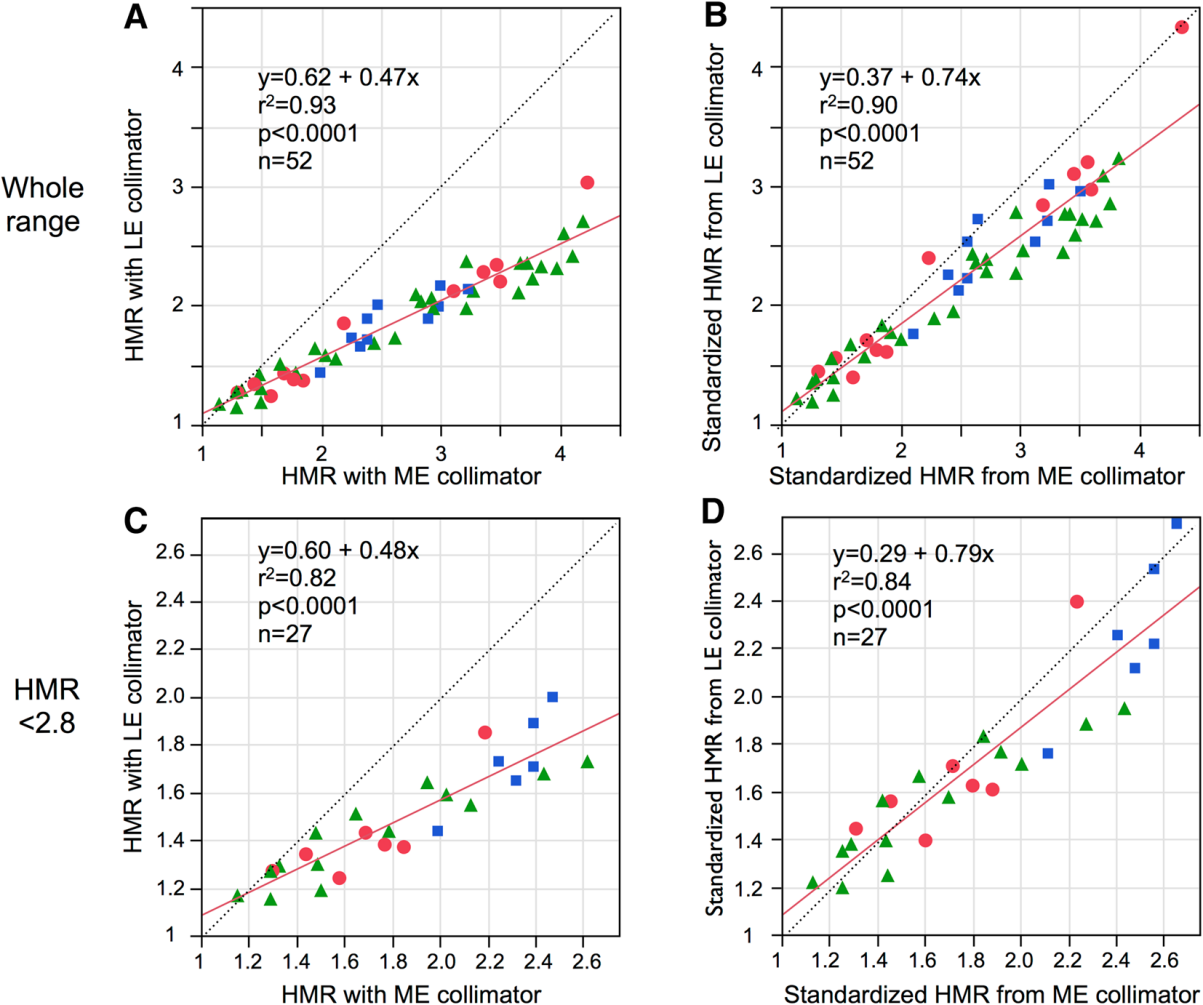
Linear regression lines of HMRs between ME- and LE- collimator types in clinical validation studies. (**A**, **B**) show the relationship before and after standardization. (**C**, **D**) show the range of HMR <2.8. The *marks* of *circle*, *square*, and *triangle* are HMRs from three hospitals. *Dotted lines* indicate line of identity

As shown in Figure 4B, D, HMRs from LE-collimator types were corrected using an experimentally determined conversion coefficient to the reference value based on the results of individual hospitals. It was converted again from the reference value to HMR_standard_ using a coefficient of *K*_standard_ = 0.88, which derived from the average coefficient of the MEGP collimator (Table 1). HMRs from ME-collimator types were similarly converted to the HMR_standard_ using *K*_standard_ = 0.88.

HMRs with the LE-collimator group showed significant underestimation; (HMR with LE-collimator) = 0.62 + 0.47 × (HMR with ME-collimator) (Figure 4A). After the correction to standardized HMRs for both LE and ME collimators, they showed comparable values below 2.5. However, underestimation of approximately 10% was observed in an HMR range of >2.5. The relationship below HMR <2.8 with ME-collimator is plotted in Figure 4C, D.

When the HMR was divided into three groups using the threshold of 2.2 and 1.6, the contingency table showed that the degree of agreement was κ 0.07 (95% confidence of interval [CI] −0.026 to 0.17, *P* = .21) and complete agreement was 25/52 (48%) between the HMRs from LE and ME types (Table 2). However, the standardized HMR to the average ME-type showed good agreement between the corrected LE-type and corrected ME types (κ 0.83, 95% CI 0.69-0.97, *P* < .0001) and complete agreement was 47/52 = 90%. A total of 22 patients (42%) were reclassified into the same risk groups after standardization.

**Table 2:** Contingency table for three HMR risk groups

LE:	High risk (<1.60)	Low risk (1.60–2.19)	Normal (≥2.20)	Total
ME				
A. HMRs measured with ME- vs LE-collimator types				
High risk (<1.60)	10	0	0	10
Low risk (1.60–2.19)	8	2	0	10
Normal (≥2.20)	0	19	13	32
Total	18	21	13	52
B. Standardized HMR converted from ME- and LE-collimator types				
High risk (<1.60)	10	1	0	11
Low risk (1.60–2.19)	1	7	0	8
Normal (≥2.20)	0	3	30	33
Total	11	11	30	52

A. Degree of agreement: κ = 0.07, *P* = .21, complete agreement = 25/52 (48%). B. Degree of agreement: κ = 0.83, *P* < .0001, complete agreement = 47/52 (90%).

## Discussion

As a number of MIBG studies have been performed using the HMR in fields of cardiology and neurology, this study focused on unifying the methods for calculating HMR. Using the calibration phantom experiments and measured conversion coefficients specific for individual camera-collimator systems, all the HMRs could be converted to standardized HMRs using the average conversion coefficient of the commonest ME types. The standardized HMRs were applicable to multiple institutions in the range of normal to low HMR values.

The difference in collimator types is one of the most important factors that affect the variation of HMRs. In particular, HMRs derived from ME-type collimators are significantly higher than those from LE-type collimators.^9^,^14^,^15^ As shown in the JSNM-working group normal databases of ^123^I-MIBG, average late HMR was 2.5 and 3.0 for LE-collimator and ME-collimator groups.^16^ The EANM Cardiovascular Committee and the European Council of Nuclear Cardiology recommended the use of the ME-collimator, which provided stable results.^8^ However, the classification into two collimator types seems to be too simple today. Collimator specifications have been modified to cover a higher energy of 159 keV of ^123^I, and the LME collimator, which is widely used in Japan, and extended low-energy collimators have become available. Even in ME types, the effects of septal penetration and Compton scatter depend on the three-dimensional structure of the collimator septa and holes. Therefore, a correction method to integrate a large variation of collimator design was sought after.

Regarding acquisition conditions, both energy windows of 20% and 15% have been used and acquisition time ranged from 3 to 10 minutes in Japan. However, in this study, although we decided on common data acquisition protocol in the phantom study, we did not compel all hospitals to use specific acquisition conditions for clinical MIBG imaging. A minor difference in conversion coefficients might have therefore been observed even with the same collimator due to acquisition conditions. Even with a single vendor’s camera and collimator combination, an SD of coefficient was 0.02-0.03 as shown in Table 1. The reasons for this variation might be explained by composite factors such as an energy window (15% or 20%), acquisition time, and back scatter from the opposite detector, SPECT couch and floor. Although fraction of the high-energy photon (529 keV) of ^123^I is only 1.4%, the effect of down scatter is complicated and troublesome.

While various methods have been proposed to improve the quantification of ^123^I tracers, including a multiple energy window method,^9^,^15^ deconvolution of the septal penetration method,^17^ and a direct empirical conversion method,^18^ the calibration phantom method has an advantage, which is demonstrated as follows. Although the simplest approach was creating a linear regression equation between measured HMRs with both LE and ME collimators, the empirical method cannot be applicable to other camera-collimator combinations or multicenter study protocols. The second approach was based on multi-window acquisition methods. However, subtraction of sub-energy window usually shows low counts, which caused errors in calculation.^14^ In contrast, the phantom-based method used in this study is considered to be a simple method to understand differences in camera and collimator systems.^10^ With this phantom, because of the homogeneous and flat distribution of the tracer, the reproducibility of HMRs was excellent when the acquisition condition was the same. It was also confirmed that in JSNM-working group databases, the HMR measured with LE-type collimators and corrected by the calibration phantom showed similar normal distribution comparable to that from ME collimators.^10^,^16^

In 225 experiments of 84 institutions, the similar collimators, for example LEHR and LEGP, did not show the same conversion coefficient, depending on the vendors. Even in the same vendor, the ELEGP collimator made from 2002 to 2004 showed significantly different values compared with that made after 2008, and the latter showed higher HMRs. This sort of modification and improvement of collimator design has been performed in companies, although the name of collimators was the same. According to the results of this study, the collimator types might be classified into 7 or 8 major subgroups. Hence, two types, namely LE and ME types, are considered a rough classification at present, and the borders of LE and ME types are obscure.

Feasibility for converting to the standard HMR was investigated in this study. In the multicenter study for differentiating dementia with Lewy bodies and Alzheimer disease, HMRs derived from LE collimators were converted to institutional ME-type comparable values.^10^ In the study, we assumed that the differences among various ME types were small, and combined distribution of HMR was improved after correction. Because we cannot use a specific collimator from one vendor as a single standard, our proposal is to use the average *K*_standard_ of MEGP collimators as the standard. With this approach, only an initial phantom experiment is required when a new camera system is installed. Although mathematical values might have been used as the standard, all institutions should change their HMRs for institutional daily practice.

The cross-calibration method can be used to apply other published studies to one’s own institution. For example, a published study used the LE collimator, and the LME collimator might be used in an individual hospital. Based on the ADMIRE-HF study showing a threshold of 1.60 for good and poor prognosis in patients with chronic heart failure using a LE-type collimator,^12^ the users of the LME collimator can assume that it is comparable to 1.91 in their institution. If the HMR of 1.77 was used to differentiate dementia with Lewy bodies and others using LE-type collimators,^19^ a threshold of 1.77 can be translated to 2.16 for users of the LME collimator. Although a number of studies using a certain threshold of HMR are valid in the similar acquisition conditions, we might be able to integrate experiences of published MIBG studies using this sort of cross-calibration.

When a prediction model is considered for predicting future mortality, current large-scale databases might be prepared using the conventional LEHR collimators.^20^ Recently, HMRs have been gradually increasing because new collimators covering ^123^I energy have become available in the nuclear medicine field. If an HMR from ME-comparable conditions is directly used for predicting mortality rate, it may underestimate the risk for future events, and the measured HMR should be converted to LE-comparable values.

## Limitations

Even after the standardization based on the phantom study, we found slight underestimation of HMRs in the range of HMR >2.5, probably due to the structural difference between the phantom and the human body. One of the important differences in human is complicated down-scatter activity from the high-energy photons coming from outside of the filed of view, such as liver, kidney, and bladders.

While complete correction from a physical point of view is ideal in a whole range, the major purpose of standardization is its use in determining a threshold for prognosis and a lower normal range. From this consideration, the most important range of HMR was around 1.6-1.7 for discriminating good and bad prognosis, and a threshold of 2.0-2.2 between normal and abnormal.^2^,^12^,^13^,^16^ In other words, the normal or higher range has no prognostic meaning. Finally, standard HMR used averaged values from 44 experiments with the commonest ME types, which may be modified when required.

## New Knowledge Gained

In terms of HMR calculation, the collimator type was classified not simply into two LE and ME types but includes various intermediate types. Using the cross-calibration phantom method, however, institutional HMRs can be converted to standardized HMRs comparable to the commonest ME-collimator. The cross-calibration method works well in the range of low and normal HMRs when classifying into main risk groups.

## Appendix

Participated institutions included Asahikawa City Hospital (Asahikawa), Bell Land General Hospital (Sakai), Chubu Medical Center Kizawa Memorial Hospital (Minokamo), Chuno Kosei Hospital (Seki), Dokkyo Medical University (Shimotsuga), Fukushima Medical University (Fukushima), Gifu Prefectural General Medical Center (Gifu), Gifu University Hospital (Gifu), Haibara General Hospital (Makinohara), Hakodate Municipal Hospital (Hakodate), Higashiosaka City General Hospital (Higashiosaka), Hiroshima Red Cross Hospital & Atomic-bomb Survivors Hospital (Hiroshima), Iizuka Hospital (Iizuka), Ikeda Municipal Hospital (Ikeda), Itami City Hospital (Itami), Japanese Red Cross Asahikawa Hospital (Asahikawa), Japanese Red Cross Ashikaga Hospital (Ashikaga), Japanese Red Cross Gifu Hospital (Gifu), Japanese Red Cross Kitami Hospital (Kitami), Japanese Red Cross Kyoto Daini Hospital (Kyoto), Jichi Medical University Hospital (Shimotsuke), Juntendo Tokyo Koto Geriatric Medical Center (Tokyo), Juntondo University Hospital (Tokyo), Juntondo University Nerima Hospital (Tokyo), Juntondo University Urayasu Hospital (Urayasu), Kanagawa Prefectural Ashigarakami Hospital (Ashigarakami-gun), Kanazawa Municipal Hospital (Kanazawa), Kanazawa University Hospital (Kanazawa), Kawasaki Hospital (Kobe), Keio Univeristy Hospital (Tokyo), Kin-ikyo Chuo Hospital (Sapporo), Konan Kakogawa Hospital (Kakogawa), Kyoto Prefectural University of Medicine - University Hospital (Kyoto), Mimihara Oimatsu Clinic (Sakai), Murakami Memorial Hospital Asahi University (Gifu), Nagaoka Red Cross Hospital (Nagaoka), Nagasaki Rousai Hospital (Sasebo), Nagoya City East Medical Center (Nagoya), National Center for Geriatrics and Gerontology (Obu), National Center for Global Health and Medicine (Tokyo), National Cerebral and Cardiovascular Center (Suita), National Hospital Organization Disaster Medical Center (Tachikawa), National Hospital Organization Kumamoto Saishunso National Hospital (Koshi), National Hospital Organization Minami Kyoto Hospital (Joyo), National Hospital Organization Okinawa National Hospital (Ginowan), National Hospital Organization Tokyo National Hospital (Kiyose), National Hospital Organization Yokohama Medical Center (Yokohama), National Kyushu Medical Center (Fukuoka), Nippon Medical School Hospital (Tokyo), Nishikobe Medical Center (Kobe), NTT Medical Center Tokyo (Tokyo), Obihiro-Kosei General Hospital (Obihiro), Ogaki Municipal Hospital (Ogaki), Okayama Kyokuto Hospital (Okayama), Okayama University Hospital (Okayama), Osaka General Medical Center (Osaka), Rumoi Municipal Hospital (Rumoi), Saiseikai Kumamoto Hospital (Kumamoto), Saiseikai Noe Hospital (Osaka), Sapporo City General Hospital (Sapporo), Sapporo-Kosei General Hospital (Sapporo), Seihoku Chuo Hospital (Goshogawara), Shizuoka General Hospital (Shizuoka), Showa General Hospital (Kodaira), Sunagawa City Medical Center (Sunagawa), Surugadai Nihon University Hospital (Tokyo), Suzuka Central General Hospital (Suzuka), Tenri Hospital (Tenri), The Cardiovascular Institute Hospital (Tokyo), The Jikei University Hospital (Tokyo), Toho University Omori Hospital (Tokyo), Tohoku University Hospital (Sendai), Tokyo Medical University (Tokyo), Tokyo Medical University Hospital (Hachioji), Tokyo Metropolitan Geriatric Hospital and Institute of Gerontology (Tokyo), Tokyo Women’s Medical University Hospital (Tokyo), Toranomon Hospital (Tokyo), Tottori University Hospital (Yonago), Toyonaka Municipal Hospital (Toyonaka), Tsukuba University Hospital (Tsukuba), Yogogawa Christian Hospital (Osaka), Yokohama City University Hospital (Yokohama), Yokohama City University Medical Center (Yokohama), and Yokohama Minami Kyousai Hospital (Yokohama).
